# Deep user identification model with multiple biometric data

**DOI:** 10.1186/s12859-020-03613-3

**Published:** 2020-07-16

**Authors:** Hyoung-Kyu Song, Ebrahim AlAlkeem, Jaewoong Yun, Tae-Ho Kim, Hyerin Yoo, Dasom Heo, Myungsu Chae, Chan Yeob Yeun

**Affiliations:** 1 Korea Advanced Institute of Science and Technology, Daejeon, South Korea; 2 Electrical Engineering and Computer Science Department, Khalifa University of Science and Technology, Abu Dhabi, United Arab Emirates; 3 Center for Cyber-Physical Systems, Khalifa University of Science and Technology, Abu Dhabi, United Arab Emirates; 4 Research Institute, NOTA Incorporated, Gangnam-gu, Seoul, South Korea; 5 Institute for Artificial Intelligence, Korea Advanced Institute of Science and Technology, Daejeon, South Korea

**Keywords:** Person identification, Multimodal learning, Multitask learning

## Abstract

**Background:**

Recognition is an essential function of human beings. Humans easily recognize a person using various inputs such as voice, face, or gesture. In this study, we mainly focus on DL model with multi-modality which has many benefits including noise reduction. We used ResNet-50 for extracting features from dataset with 2D data.

**Results:**

This study proposes a novel multimodal and multitask model, which can both identify human ID and classify the gender in single step. At the feature level, the extracted features are concatenated as the input for the identification module. Additionally, in our model design, we can change the number of modalities used in a single model. To demonstrate our model, we generate 58 virtual subjects with public ECG, face and fingerprint dataset. Through the test with noisy input, using multimodal is more robust and better than using single modality.

**Conclusions:**

This paper presents an end-to-end approach for multimodal and multitask learning. The proposed model shows robustness on the spoof attack, which can be significant for bio-authentication device. Through results in this study, we suggest a new perspective for human identification task, which performs better than in previous approaches.

## Background

Recognition is an essential function of human beings. Humans easily recognize a person using various inputs such as voice, face, or gesture. Thus, when engaging with deep learning, multi-modality needs to be taken into account instead of single modality.

Using multi-modality has many benefits including noise reduction. As any single modality can be easily contaminated due to powerline, EMG, channel noise, electrodes, and motion artifact, it is difficult to build a single modal recognition algorithm. For multimodal model, if a modality has a low signal to noise ratio, it can be compensated by another modality, so the overall system performance is maintained. Additionally, in the training phase, correlated features among modalities can be trained, and this may lead to a better performance. Moreover, when compared to training three independent models on each modality, it is more efficient to train a single multimodal model.

In this study, we mainly focus on DL model to get better result. Previously, traditional machine learning methods were engaged for person identification. However, ML contains pipeline of hand-crafted feature extraction followed by classification model such as k-NN, random forest, and etc. Since the feature is hand-crafted, there is no guarantee that only informative features are extracted. Using DL, the choice of feature extraction and the learning model is far less restricted comparing to ML.

In the real world, the ECG from a person may vary because of several factors such as heart rate, sickness, etc. If the model is trained using multitask learning, the model can focus only on the target task, and ignore factors that are of no interest. In addition, there may be noise in the procedure to collect data. Therefore, it is necessary to develop a robust model architecture, considering generalization.

In this paper, we introduce our user identification model which uses three biometric data: ECG, face, and finger. Moreover, this model is designed for solving supplementary tasks such as gender classification. Finally, to use multimodal biometrics, it fuses features from each biometric data and is trained with a deep neural network; thus, it can be robust on noisy data.

## Related Work

Recent studies have proposed new approaches to enhance system security. They have adopted the method of combining two or more multiple biometric data to identify or certify users. These techniques have demonstrated that extracting features from multi-modality and fusing them is effective in increasing the accuracy of the verification [1]. Sara and Karim emphasized the effects of adopting multi-modality in their work, which showed that the performance was raised from 82.1% (only palmprint) and 89% (only ECG) to 94.7% (multi-modality; palmprint and ECG) [2].

A virtual multimodal database is widely used in multimodal biometric research [3–5]. A virtual dataset can reduce wastage of time and the cost of data collection. Let us assume that we have two databases which are mutually exclusive on modality and subjects. Then, the virtual multimodal database is constructed by matching subjects in the two databases to allow a single person to obtain information on both modalities. This could be performed based on the assumption that different biometric data traits of the same subject are independent [6]. There is no public multimodal database which consists of ECG, face, and fingerprints extracted from the same person.

## Methods

**Fig. 1:**
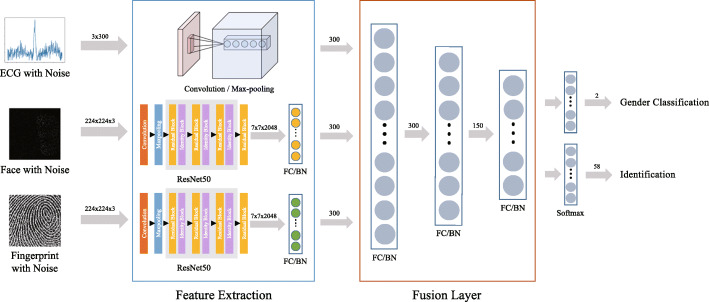
The overall network architecture This network architecture is largely divided into three parts, feature extraction which transforms the input into embedding space, fusion layer which combines features from each modality, and task layer which performs the task. The figure excluding data sample images is our own illustration

As shown in Fig. 1, the network architecture is largely divided into three parts: feature extraction, which transforms the input into an embedding space; fusion layer, which combines features from each modality; and task layer, which performs the task. First, feature extraction is performed on each modality using a different method as each modality has different characteristics. The datatype of fingerprint *x*_*p*_ and face *x*_*f*_ is a static image while ECG *x*_*e*_ is a temporal biological signal. It is well known that a classifier trained on large database can be used as a general feature extractor [7].

We used ResNet-50 for extracting features from *x*_*f*_ and *x*_*p*_. Additionally, a fully connected layer is used for matching the number of dimensions to that of ECG. While CNN architecture followed by max pooling is designed for *x*_*e*_ for ECG, the extracted feature does not rely on the temporal axis. Second, the fusion layer takes concatenated features from the feature extraction module as an input. We use neural networks for the fusion layer. As the data propagates, the information of each modality is mixed up to decrease a loss. Finally, the task layer takes the output of fusion layer as input.

The model calculates the loss with categorical cross-entropy for user identification, and binary cross-entropy for gender classification. For multitask experiment, the joint loss is decided from these two losses by adding with same weight.

### ECG preprocessing

We cropped a total of 300 data values before and after R peak of the signal to generate a QRS complex vector. After this procedure, min-max normalization is applied to each QRS complex. We can get at least 15 QRS complexes from each record. As mentioned earlier, because we have the intuition that temporal information between QRS complexes is less important, we extract three QRS complexes for one input from a record. In conclusion, one input sequence has three timesteps, which means that three QRS complexes are grouped to an input sequence.

### Feature extraction

Residual Network (ResNet [8]) is a widely used feature extractor for images. ResNet is trained on the ImageNet database, which is the largest database for object classification. We utilize ResNet50 as a feature extractor for face and fingerprint. We use average pooling of feature (7 ×7 ×2048).

Although there is a model based on the LSTM architecture for user identification [9], we have used the CNN architecture as a feature extractor with the intuition that temporal information between QRS complexes is less important. The CNN model has a 1D convolution layer which takes a (batch size, time step, 300) tensor as input and outputs a (batch size, time step, 300) tensor. After the 1D convolution layer, 1 max pooling operation is used. The extracted ECG biometric feature is concatenated with 2 feature vectors from other modalities.

### Feature fusion

There are many fusion methods. In terms of improving the model performance, it is very difficult to properly fuse three different modalities at the input level. We compare the performance between score level fusion and feature level fusion. The three methods of score level fusion, which are sum, product, and max rule, were tested. At the feature level, the three extracted features from each modality are respectively normalized and then concatenated as the input for the model. In our model design, when the specific biometric data is not given, the feature value for corresponding modality is processed as 0 and excluded from BN layers. Thus, we can change the number of modalities used in a single model.

### Classification

Our user identification system decides the class with the highest probability for each test sequence. For this reason, softmax activation function is used; it takes the output of each class and converts them into their respective probabilities using the following equation:

1 $$ Softmax(y_{i}) = \frac{exp(y_{i})}{\sum_{j}exp(y_{j})}  $$

where *y* is the output of the network of which dimension is same to the number of classes. *y*_*i*_ is the *i*^*th*^ element of the vector y. This function applies the standard exponential function to each element *y*_*i*_ of the input *y* and divides by the sum of all these exponential terms, ensuring that the probabilities sum to one; the target class will have the highest probability. For user identification and gender classification, we use the cross-entropy loss as the cost function during the model training. Cross entropy or log loss compares the output probabilities of predictions with the one-hot encoded real-labels. It is defined as:

2 $$ H(y,\hat{y}) = -\sum_{i}y_{i}\,log\,\hat{y}_{i}.  $$

where *y* is the output distribution and $\hat {y}$ is the original distribution. The negative log of the output probabilities penalizes the wrong predictions: the loss is high when the probability of the predicted class diverges from the actual label. This loss function is not symmetric i.e. $H(y,\hat {y}) \neq H(\hat {y}, y)$ log is taken only of predicted probabilities

### Joint loss

For jointly training the network to deal with multiple tasks simultaneously, we use joint loss that combines binary cross entropy for gender classification (*L*_1_) and categorical cross entropy for user identification (*L*_2_). We set final loss function for model training with a weighted sum of two losses. The formula for each loss is shown as below. The optimal values of *w*_1_ and *w*_2_, where both are 0.5 and 1 in total, is found from sweeping these hyperparameters with grid-searching method.

3 $$ L_{1} = -y\,log\,\hat{y} - (1-y)\,log\,(1-\hat{y})  $$

4 $$ L_{2} = -\sum_{i}y_{i}\,log\,\hat{y}  $$

5 $$ L_{joint} = w_{1} \times L_{1} + w_{2} \times L_{2}.  $$

### Dataset

This subsection presents our experiments for verifying the superiority of the proposed model using the accuracy metric. Three experiments were conducted in this research. The first experiment compared single modality and multimodality for multitask learning (gender classification and user identification). The second experiment was a comparison between multitask learning and single task learning by the multimodal multiple biometric data, which consisted of ECG data (ECG-ID database [10, 11], PTB database [10, 12]), face data (Face95 [13]), and fingerprint data (FVC2006 [14]). Finally, we add noise to the data to verify its robustness. Especially, we assumed that the face and fingerprint are used as supplementary multiple biometric data, so we introduced severe noise to these two modalities.

**ECG** For ECG, we combined two different dataset, ECG-ID database and PTB database, both of which can be downloaded from Physiobank in Physionet. By using multiple dataset for the biometric data, the model can work regardless of where the signals are measured. The ECG-ID database contains 310 recordings from 90 subjects (44 males and 46 females, aged from 13 to 75 years). Each recording was measured by Lead I, recorded for 20 seconds, and digitized at 500 Hz with 12-bit resolution over a nominal 10 mV range. The PTB ECG database contains 549 records obtained from 290 subjects (209 males and 81 females, aged between 17 and 87). Each record includes 15 simultaneously measured signals: the conventional 12 leads (i, ii, iii, avr, avl, avf, v1, v2, v3, v4, v5, v6) together with the 3 Frank lead ECGs (vx, vy, vz). Each signal is digitized at 1 kHz with 16-bit resolution over a range of ±16.384 mV. To synchronize the sampling frequency with that of the ECG-ID database, all 15 ECG signals from the PTB ECG database was re-sampled by 500 Hz.

**Face** The Faces95 dataset contains 1,440 images (20 images per individual), obtained from 72 individuals (male and female subjects). The individuals are mainly undergraduate students. The image resolution is 180 ×200 pixels (portrait format). During the collection of data, the subjects took a step forward towards the camera. This caused Head scale variation, lighting variation, and translating position of the face in the image. Lastly, there are some expression variations. However, there is no hairstyle variation.

**Fingerprint** The FVC2006 contains 7,200 images obtained from 150 individuals. These 7,200 images consist of 4 different sensors, which are Electric Field sensor, Optical Sensor, Thermal sweeping Sensor, and SFinGe v3.0 (1,800 images per sensor). Each database is divided into two subsets, A and B. For subsets DB1-A to DB4-A, each subset contains 140 subjects with 12 images, yielding 1,680 images. For other subsets DB1-B to DB4-B, each subset contains 10 subjects with 12 images, yielding 120 images. The image resolution of the four sensors is 96 ×96 pixels, 400 ×560 pixels, 400 ×500 pixels, and 288 ×384 pixels.

**Virtual Dataset** We generated 58 virtual subjects to reduce the variability of the given multimodal database. Each virtual subject is labeled with a person ID and its sex based on ECG and face data. As the subjects of Face95 are mainly undergraduate students, we assumed that they were between their teens and thirties. The gender label is achieved by two annotators fully in agreement. Then, using two criteria: the gender that was labeled on the face data, and the age between teens to thirties, we select a proper sample that considers age and sex from the ECG dataset and match it with face data. Also, we used a fingerprint image from DB1_A of the FVC2006 database, which are collected with one scanner.

We assigned it randomly to a virtual subject that was already assigned ECG and face data. For face and fingerprint, we augment the data with rotation, translation and cropping method. For ECG, we combine three QRS complexes from 15 segments. Therefore, there are at least 400 data in each biometric data for one virtual subject and these samples are made into 400 triplets, which consists of ECG sample signal, face sample image, and fingerprint sample image. From this combined dataset, the model randomly chooses 80% of the database as training sets, and tests with the remaining data. We apply k-fold cross-validation to train with training sets whose k is set to 8. In other words, we use 70% of data in training session, 10% of data for validation and the remaining 20% of data for testing the performance of our model.

## Results

**Fig. 2:**
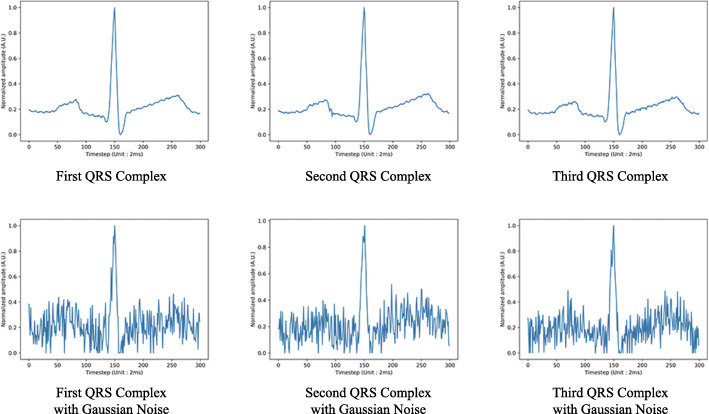
Noise to ECG input data For ECG, we add Gaussian noise for serials of three normalized QRS complexes whose mean is zero and standard deviation is 0.1

**Fig. 3:**
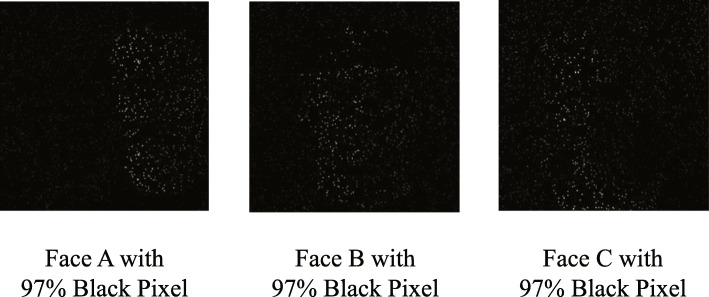
Noise to face input data For face, we select 97% of facial image pixels and fill with black. The raw face image is not included to follow conditions of using public data

**Fig. 4:**
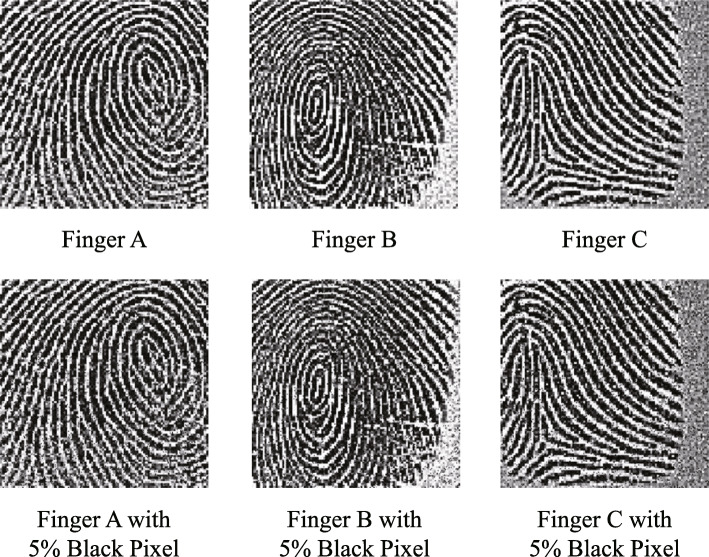
Noise to fingerprint input data For finger, we select 5% of finger image pixels and change the color to black

**Unimodal and Multimodal Model** In this experiment, we add noise for better generalization and discrimination between models. For ECG, we add Gaussian noise for serials of three normalized QRS complexes whose standard deviation is 0.1. The changed data are shown in Fig. 2. For face, we select 97% of the face image pixels and fill it with black in Fig. 3. For fingerprint, we select 5% of finger image pixels and change the color to black as Fig. 4. In Table 1, the accuracy is shown to be more precise for user identification when all three modalities are used (98.28%) than in all other cases. Additionally, for gender classification, models with all three modalities show better results (97.70%) than models depending on a sole modality.

**Table 1:** Accuracy for the Combination of Modality Usage

Modality			Accuracy (%)	
ECG	Face	Finger	ID	Gender
O			77.49	91.95
	O		76.44	88.51
		O	83.91	90.80
O	O		95.98	93.68
O		O	94.83	95.40
	O	O	96.55	94.83
O	O	O	98.28	97.70

When using all three biometric data for each task, it performs the best result for both identification and gender classification tasks

From this result, we find that we can determine subjects well with the feature-level fusion model even if multiple inputs are noisy. Comparing multimodal models of two modalities with those of three modalities, we see that decisions from multiple biometric data are much more accurate than those from limited modalities.

**Single Task and Multitask** For the experimental result of multitask learning shown in Table 2, we see better results (98.97% accuracy) in the multitask model for the identification task. It surpasses the performance of a single task model (98.28%). However, for gender classification, it shows better results when using the single task model (97.70%). In this situation, we considered the model performance as the averages of both tasks. So, even if we cannot get the best accuracy for gender classification, the performance of the identification task improved. Considering the efficiency in both time and space, the proposed multitask model is much more efficient because its predictions are as good as the single task model while the time required for model training is reduced by half.

**Table 2:** Single task model and multitask model

Task		Accuracy (%)	
ID	Gender	ID	Gender
O		98.28	-
	O	-	97.70
O	O	98.97	96.55

When dealing with multiple tasks, it is more accurate in identification task. Since the model trains for both identification and gender classification task, the process to classify gender can assists in identifying human being. For gender classification task, our multitask model seems a bit less accurate than that in single task model. However, considering the number of parameters for models, the proposed model is efficient

**Robustness to Noisy Data** As shown in Table 3, after adding noise to all multiple biometric data, the highest performances for the two tasks, identification and gender classification, are 98.97% and 96.55%, respectively. These experiments illustrate that the performance of multimodal is better than the accuracy of single modality even though noise is included. The result of using clean modalities also indicates similar aspects when using noisy modality. In identification, multimodal has an accuracy of near 100%. In gender classification, three multimodal provided an accuracy of 99.43%. From this experiment, we see that the difference in performance of three multimodal with noise was 1.03%. This illustrates that the proposed architecture is significantly reliable and robust to noises.

**Table 3:** Multimodal and multitask test for noisy input

Modality			Noise	Accuracy (%)	
ECG	Face	Finger		ID	Gender
O	O		O	94.83	95.02
O		O	O	93.68	95.21
	O	O	O	95.21	92.91
O	O	O	O	98.97	96.55
O	O			100.0	100.0
O		O		98.85	96.55
	O	O		100.0	98.85
O	O	O		100.0	99.43

From this experiment, not only does the clean modalities provides near 100% in two tasks, but in the case of noisy modalities, the feature fusion of three modalities yields 98.97% in identification. This result shows that the model architecture is robust to noises

**Fusion Methods for Feature Maps** Unlike to single-modality, multi-modality used fusion technique to concatenate biometric characteristic. This methodology has the advantage of reducing individual aspects of each modality so it improves reliability and accuracy of system using biometric data. In fusion method, while feature level fusion is a method before matching which consolidate features of modalities to one feature vector, score-level fusion is a method after matching which calculate the degree of similarity using rules (sum, product, max) from each output of modality and reach the final output vector. In Table 4, the overall performance obtained in comparison between feature-level fusion and score-level fusion in identification and gender classification shows the result of high accuracy using fusion method. In addition, to test score-level fusion with two different cases, we experiment which rules can achieve the highest accuracy among three different rules such as sum, product, max and the performance depending on the weight of modality.

**Table 4:** Comparison between feature-level fusion and score-level fusion

Fusion Level	Rule	Weight			Accuracy (%)	
		ECG	Face	Finger	ID	Gender
Feature	-	-	-	-	98.97	96.55
Score	Sum	0.33	0.33	0.33	98.27	99.42
		0.50	0.25	0.25	98.85	99.42
		0.25	0.50	0.25	98.85	99.42
		0.25	0.25	0.50	97.70	99.42
Score	Product	0.33	0.33	0.33	96.55	89.08
		0.50	0.25	0.25	95.98	89.66
		0.25	0.50	0.25	93.10	89.08
		0.25	0.25	0.50	93.68	87.36
Score	Max	0.33	0.33	0.33	89.66	89.66
		0.50	0.25	0.25	89.66	87.93
		0.25	0.50	0.25	89.66	86.21
		0.25	0.25	0.50	89.66	87.36

From this experiment, the feature fusion of three modalities shows the best performance in user identification task. For gender classification task, the score-level fusion shows the best performance regardless of weights in each physiological data. This result shows that feature-level fusion method shows good performance even without adjusting weight

In feature-level fusion, performances of two tasks are 98.97% and 96.55%, which the accuracy of the two tasks is over 96%. Also, in score-level fusion, the accuracy is 98.85% based on sum rule for identification and 99.42% for gender classification. It illustrates that the performance of both tasks can achieve the accuracy of over 98%. The accuracy of score-level fusion with sum rule is improved by up to 2.87% compared feature-level fusion for gender classification. In other words, sum rule provides the highest accuracy among rules from score-level fusion because sum rule is vigorous from high noise. In score-level fusion, we also experimented with changing weights statically for each modality. To emphasize specific modality among three different modalities, we assigned a weight of 0.5 to one modality and weight of 0.25 to the rest of the two modalities. The result of this experiment explains that the highest accuracy among every case is obtained when weight of ECG is higher or equal than other modalities. From this result, ECG is seen as modality which contribute more to improve performance than the rest of the two modalities, face and fingerprint.

## Discussion

As the number of devices is increasing for personal identification through multi-modalities, using single biometric information may cause a security problem. This study has a limitation that it performs one additional task and deals with only three biometric information, ECG, face, and fingerprint. Also, the data that we used in this research is not big enough (with 58 subjects), so it However, since the feature extracting method is much more independent on the type of data than previous studies, it is fully possible to substitute other multiple biometric data. In addition, the fusion in feature layer enables the model to learn and infer even when there are N modalities. Taken together, applying multiple biometric data in a single model not only has the advantage of using parameters efficiently, but also enhances security because these can be processed on a single device.

In the future, some supplementary points should be considered to make the proposed model achieve a similar performance when missing one of three biometric data. In addition to user authentication, we are working on improving our model to work with missing modalities. These results would help indicate that it can be deployed in real-world applications with high security. Additionally, the whole network can be trained using an end-to-end approach with more modalities. Lastly, the technique used for the fusion of multimodal data can be further improved by employing the attention model to choose proper modality or features that are expected to play an important role in the given sample.

## Conclusions

This paper presents a novel approach for multimodal multitask learning which is robust to noise. ECG, face image, and fingerprint dataset are used for multimodal learning. Additionally, this research focused on user identification and gender classification. The proposed model achieves higher accuracy for both tasks. In addition, the proposed model shows robustness on the spoof attack problem that confronted most models based on single modality. With the results of the experiments, we insist that the performance of the proposed model for user identification and gender classification are better than in previous approaches.

## Data Availability

The ECG dataset analysed during the current study are available in the Physionet ECG-IDDB repository(physionet.org/content/ecgiddb/1.0.0/) and PTB repository(physionet.org/content/ptbdb/1.0.0/). The face dataset analysed during the current study are available in the Libor Spacek’s Facial Images Databases repository (cmp.felk.cvut.cz/ spacelib/faces/) under conditions of use. The fingerprint dataset analysed during the current study are available in the FVC2006: the Fourth International Fingerprint Verification Competition repository(bias.csr.unibo.it/fvc2006/). The dataset used and/or analysed during the current study are available after registering in the website.

## Abbreviations

DL: Deep learning
ResNet: Residual Network
ECG: Electrocardiogram
EMG: Electromyography
ML: Machine learning
CNN: Convolutional neural network
LSTM: Long short-term memory
BN: Batch normalization
PTB: Physikalisch-Technische Bundesanstalt
SFinGe: Synthetic fingerprint generator
