# Correction to: Deep user identification model with multiple biometric data

**DOI:** 10.1186/s12859-020-03746-5

**Published:** 2020-10-26

**Authors:** Hyoung-Kyu Song, Ebrahim AlAlkeem, Jaewoong Yun, Tae-Ho Kim, Hyerin Yoo, Dasom Heo, Myungsu Chae, Chan Yeob Yeun

**Affiliations:** 1 Korea Advanced Institute of Science and Technology, Daejeon, South Korea; 2 Electrical Engineering and Computer Science Department, Khalifa University of Science and Technology, Abu Dhabi, United Arab Emirates; 3 Center for Cyber-Physical Systems, Khalifa University of Science and Technology, Abu Dhabi, United Arab Emirates; 4 Research Institute, NOTA Incorporated, Gangnam-gu, Seoul, South Korea; 5 Institute for Artificial Intelligence, Korea Advanced Institute of Science and Technology, Daejeon, South Korea

**Correction to: BMC Bioinformatics 21, 315 (2020)**

**https://doi.org/10.1186/s12859-020-03613-3**

Following publication of the original article [1], it was reported that the article entitled “Deep user identification model with multiple biometric data” was published in the regular issue of this journal instead of in the supplement issue.

The details of the supplement in which this article ought to have been published are given below:

**About this supplement**

This article has been published as part of BMC Bioinformatics, Volume 21 Supplement 5, 2020: Proceedings of the 13th International Workshop on Data and Text Mining in Biomedical Informatics (DTMBIO 2019). The full contents of the supplement are available at https://bmcbioinformatics.biomedcentral.com/articles/supplements/volume-21-supplement-5.

The publisher apologises for any inconvenience caused.
